# Surgical Club of South West England—Abstracts of Papers

**Published:** 1985-01

**Authors:** 


### Surgical Club of South West England

### Autumn Meeting held at Exeter, 19th and 20th October 1984

### A NEW DEVON POLYP SYNDROME

**Keith Vowles, Royal Devon and Exeter Hospital, Exeter**

This first report of recurrent multiple inflammatory fibroid polyps occurred in three generations of a Devon family; in the grandmother first at the age of 59 years, in the mother first at the age of 35 years, both in Tiverton, and in the granddaughter first at the age of 22 years, three years after emigrating to New Zealand. Only one female in each generation has been affected, in a direct line of descent. The grandmother has had nine polyps resected over eleven years, the mother seven over eighteen years and the granddaughter six over six years, the presentation in most cases being due to intestinal obstruction from intussusception. None of the patients or their relatives are known to have any allergies, dietary fads or gastrointestinal infections. Comparison with the known familial gastrointestinal polyposis syndromes shows that in colonic polyposis, Gardner's syndrome, Peutz-Jegher's syndrome and juvenile polyposis each is inherited as an autosomal dominant and in each case there is an association with neoplasia, while the rare Turcot syndrome is an autosomal recessive. A genetic factor which is probably polygenic and multifactorial in nature is likely to operate in this unique family. Chromosome studies have been normal in two of our patients, and no cancer risk has been identified. Conventional histology, electron microscopy and immunohistology suggest that the lesion is a self-limiting proliferation of histiocytes, the initiating event or stimulus remains unknown.

### RESTORATIVE PROCTOCOLECTOMY WITH ILEAL PELVIC RESERVOIR

**N. J. McC. Mortensen, Bristol Royal Infirmary**

Whilst an ileostomy for life is an acceptable alternative to the misery of severe ulcerative colitis, it is quite a burden for the patient coming to colectomy because of premalignant change or for polyposis coli and is not without a psychosocial cost.

The first ileostomy procedures developed once a suitable ileostomy applicance had became available in the late 1940's. At the same time the first patients with an ileoanal anastomosis were reported, but the procedure never became popular because of frequency and perianal excoriation. Procto-colectomy and ileostomy then became the standard operation, although a minority of suitable patients were offered an ileorectal anastomosis. Kock then introduced the continent ileostomy in which a reservoir formed from ileum together with a nipple valve allowed the patient to intubate and empty the reservoir at intervals. Although early technical problems have been ironed out, the procedure has not become widely used.

In 1976 Parks carried out the first pelvic pouch operation obviating the need for a permanent ileostomy. After a standard colectomy the rectum was mobilised close to the bowel wall to avoid damage to the pelvic nerves and divided just above the pelvic floor. The remaining diseased mucosa was removed from below by stripping it off the underlying muscle leaving a tube complete with mesorectum. A reservoir was constructed from terminal ileum and sewn to the anal canal inside the muscular tube. A covering ileostomy allows for safe healing and is then closed 2 months later. Parks reported the results of 'S' shaped reservoirs in 66 patients, but a disappointing 53% could not empty the pouch spontaneously. However, 65% were completely continent, and only 3 patients have asked to have the reservoir removed. Since this pioneering work a further 320 procedures have been reported worldwide and technical modifications have both made the procedure simpler to carry out and improved function. Recent patients can expect a stool frequency of 4–6 spontaneous bowel actions per day, with complete continence. The major failures have occurred in patients with Crohns disease which is a contra indication to the procedure.

Restorative proctocolectomy with ileal pelvic reservoir is now a standard procedure which may be offered to patients with familial polyposis or ulcerative colitis and is likely to become more widely applied. The first successful preganancy and delivery by Caesarian section in a patient with a pouch was reported in 1983.

### TOTAL FUNDOPLICATION GASTROPLASTY FOR PEPTIC OESOPHAGEAL STRICTURE

**K. M. Pagliero, Royal Devon and Exeter Hospital, Exeter**

There are many possible options in the surgical management of peptic stricture of the oesophagus. In view of the high reported mortality for resection I

prefer conservative operation and I am pleased to report that in the last 5 years that I have not encountered an undilatable stricture which I largely attribute to the employment of Maloney mercury filled flexible bougies. Of the many conservative operations I am persuaded that those which are subdiaphragmatic are to be preferred. I have come to believe that in all cases of peptic stricture the oesophagus is shortened and I, therefore, lengthen the oesophagus by performing a Collis gastroplasty with which I combine a total fundoplication to control gastro-oesophageal reflux.

I present 29 cases of total fundoplication gastroplasty who have had a follow up of 1–5 years. All except one patient has been relieved of dysphagia and investigation has confirmed that in that failure the fundoplication had become undone and gastro-oesophageal reflux had returned. In this one patient it was thought at the time of the operation that there was tension in the repair due to an inadequate length of gastroplasty and that this was probably the underlying reason why the fundoplication has become undone. Of the remaining satisfactory patients four needed one further dilatation in the postoperative period; two had temporary gas bloat; one had mild permanent gas bloat and two had persistent mild post-thoractomy discomfort. The important feaures of the operation it is suggested, are the creation of a generous length of gastroplasty to eliminate tension on the repair, to ensure that the fundoplication only encloses the lower 2 cm of the neo-oesophagus and to ensure by adequate division of short gastric arteries that an extremely loose fundoplication be constructed. Total fundoplication gastroplasty has proved so far to be a very reasonable option in the treatment of dilatable peptic oesophageal stricture.

### IDIOPATHIC SMALL BOWEL ULCERATION

W. E. G. Thomas, Bristol Royal Infirmary

Non-specific ulceration of the small bowel is rare, the diagnosis is commonly overlooked and seldom established before operation. This condition was first described by Baillie in 1795, and later defined by Grasman in 1925 as 'sharp-bordered, solitary ulceration with no surrounding inflammation, of unknown cause, indefinite pathogenesis, and an acute or chronic course'. Our understanding of these curious ulcers has advanced very little in the ensuing years, and delay in diagnosis can cause the patient several years of unnecessary morbidity.

In Bristol six patients (four male, two female) developed non-specific small bowel ulceration between the ages of 4 months and 76 years, with the length of history varying from one week to 33 years. Early reports of this condition describe a high incidence of perforation, but none were seen in this series. Five patients presented with gastrointestinal haemorrhage, occult in three resulting in iron-deficiency anaemia, but with melaena in two, and was so profuse in one patient that it required emergency surgery. Four patients presented with mild obstructive symptoms of abdominal pain and distension, but physical examination was normal in all patients apart from signs of anaemia.

All patients underwent multiple investigations, but the most productive were visceral angiography and small bowel enemas. At laparotomy the ulceration was found to be jejunal in two cases and ileal in four. In two patients the ulcer was solitary, but was multiple in four with 2–6 separate ulcers. Surgical excision with end-to-end anastomosis was curative in all but one case, in whom recurrent ulceration has required three resections and repeated blood transfusions. Histology is characteristic but non-specific. There is usually a low grade inflammatory response with patchy pyloric metaplasia and local hyperplasia of the muscularis mucosae.

In conclusion diopathic small bowel ulceration is rare and of unknown aetiology. It is seldom diagnosed preoperatively and delay is common. The clinical presentation is changing and haemorrhage is now the most common feature. A small bowel enema is the most useful investigation and following surgical excision, the prognosis is usually good.

### CONTINUOUS HOME INFUSION CHEMOTHERPY

C. Giles Rowland, Royal Devon and Exeter Hospital, Exeter

The majority of common solid tumours are not particularly responsive to conventional bolus chemotherapy. In view of their size, long doubling time, heterogeneity and number of cells in a resting phase it is perhaps not surprising that there is very little exposure to cytotoxics especially in view of the short half lives of most of the present agents. Tumour exposure can be increased by prolonged infusion of relatively low doses of drugs for anything from 24 to 100 days.

To date some 80 patients with advanced evaluable cancers have been treated by home continuous infusion in Exeter. Silastic subclavian lines are inserted by the anaesthetist and so far we have a less than 5% complication rate. The lines are used only for drug delivery and not for taking or giving blood products. A syringe driver is worn in a holster and the patient is supplied with a seven day supply of drug preloaded into the syringes. We have studied a number of agents including Adriamycin, Epirubican, Ifosfamide, Fluorouracil and Cis Platinum and in all cases have noticed a marked reduction in toxicity particularly with regard to nausea and vomiting. The drug system works well and there is excellent patient compliance.

Although the response analysis is not the primary objective of this early study, significant responses have been observed in melanoma, breast cancer, lung cancer and colon cancer. Ongoing studies will help further to define the role of infusion therapy in cancer treatment.

### FLUOROURACIL IN CHEMOPROPHYLAXIS OF COLORECTAL CANCER: RESULTS OF A CONTROLLED CLINICAL TRIAL

**T. T. Irvin, Royal Devon and Exeter Hospital, Exeter**

It has been reported that short-term adjuvant therapy with low doses of fluorouracil (5-FU) results in improved survival in patients undergoing radical surgery for colorectal cancer. However, such reports are based on the use of historical controls, and there have been no controlled clinical trails of such therapy.

The value of short-term chemoprophylaxis with 5-FU was examined in a randomized prospective clinical trial in 128 patients undergoing radical surgical resection of primary colorectal cancer. Group 1 (63 patients) received 5-FU in two courses four and eight weeks after surgery and Group 2 (65 patients) received no chemotherapy. The two groups were well matched with regard to age, sex, Dukes' staging, and other clinical parameters. The average duration of follow-up is 5.6±0.81 years in Group 1 and 5.8±0.65 years in Group 2. Twenty-eight patients have died in each group, and recurrent disease was present in 26 patients in Group 1 (41.2%) and in 22 patients in Group 2 (33.9%). There is no significant difference in the incidence of recurrent disease of the mortality in the two groups, and this controlled trial has failed to confirm that short-term 5-FU therapy is of significant value in the chemoprophylaxis of colorectal cancer.

### THE ROLE OF THE PATHOLOGIST IN THE CLINICAL MANAGEMENT OF TUMOURS OF THE LIVER

**P. P. Anthony, Royal Devon and Exeter Hospital, Exeter**

A multiplicity of different tumours arise in the liver and many more metastasize to it. Accurate diagnosis and advice on matters likely to affect clinical management are the province of the histopathologist. Four problems arise frequently.

(1) A reliability score can be presented for histology versus cytology for individual tumours. Most benign tumours, bile duct papillomatosis excepted, require a sizeable biopsy of excision for accurate diagnosis. Liver cell carcinoma cannot, at present, be diagnosed on fine needle aspiration. Primary and secondary adenocarcinomas cannot be distinguished reliably. In many tumours, immunohistology and electron microscopy are necessary for a precise diagnosis.

(2) Important subtypes of primary tumours have been identified in recent years that carry a better prognosis and/or are more amenable to resection.

(3) It is possible to indicate, with a reasonable degree of certainty, which tumours are likely to be solitary or multiple, based on information from histopathological examination.

(4) The source of a metastasis can be established with an increasing degree of confidence, either from unique morphological features or from cell marker studies.

The presence of a malignant tumour in the liver need no longer be considered as an inevitable death warrant for the patient.

### PRIMARY TREATMENT OF BREAST CARCINOMA IN THE ELDERLY

**C. R. H. Penn, Royal Devon and Exeter Hospital, Exeter**

A consecutive series of 90 cases of carcinoma of the female breast in patients aged 70 years or over was reviewed. These patients were treated over a five-year period with a minimum follow-up interval of four years.

Patients in Stages I and II receiving conventional local treatment showed an effectively identical survival pattern to the general population with a median survival in excess of seven years. Recurrence-free survival in this group was five years.

Patients in Stage III showed a median survival of approximately three years and a recurrence-free survival of two years, whether treated with Tamoxifen alone or with conventional local treatment.

Tamoxifen was advocated as an acceptable and less toxic alternative to conventional local treatment in Stage III but not in patients in good general condition under 85 with early disease.

Statistical evaluation where the 'normal' population has a high mortality present difficulties and the co-operation of Dr. P. C. R. Taylor, of the Institute of Biometry, of the University of Exeter is gratefully acknowledged.

### EXETER EXPERIENCE WITH RENAL TRANSPLANATION

**Michael Golby, Royal Devon and Exeter Hospital, Exeter**

Renal transplantation started in the South West in Exeter in 1968. Since that time 291 patients have been treated for renal failure: 124 transplants have occurred in 118 patients (63% graft function at three years).

On reviewing the operations undergone by 89 patients (100 transplants), who have had all their treatment for renal failure in Exeter, it was found they has experienced 357 major operations and over 236 lesser operations.

From EDTA returns we know that about 19% of patients suffer renal failure from surgically treatable conditions delaying dialysis for many years. Preceeding disease necessitated nephrectomy in 20% of recipients: 30% of recipients required transplant nephrectomy: 35% of recipients required re-exploration of the graft at sometime and another 10% of patients had other major urological conditions making this the third largest group of operations.

Dialysis, steroid and parathyroid bone disease accounted for 8% of operations. Laparotomies either for general surgical conditions or omentectomy and Tenchcoff catheterisation occurred in 14% of patients. Graft implantation was the second largest group of operations, the largest being those for vascular access. The total Exeter experience of AV fistulas is: forearm 183; brachial 42; leg 5; Gortex/Bovine/hemasite button 9; jugular catheters 2.

Minor infective conditions were made worse by steroids, diabetes and low residue diet aggravated the incidence of piles. Other unrelated surgical conditions occurred in this group without increased incidence.

### ORGANISATION OF ORGAN PROCUREMENT SERVICE IN THE SOUTH WEST REGION

**Hany Riad, Royal Devon and Exeter Hospital, Exeter**

In order to increase the number of cadaveric donors we need to enhance public awareness, and encourage clinicians to provide more donors and to diagnose brain death.

The object of increasing the number of donors is the main reason for appointing Transplant Co-ordinators. The majority of them are ex-renal nurses, and the rest are perfusion technicians or administrators. Few are medically qualified. Apart from publicity, a Transplant Co-ordinator would ensure that legal aspects of organ donation are met, and would provide feed-back information to donating hospitals, and also brief information to the donors family.

In Exeter between July 1977 and June 1983 kidneys were retrieved from only 43·7% of potential donors.

The reasons for not harvesting organs from the rest were: sepsis, circulatory failure, poor renal function, extremes of age, permission refused, failure to seek permission, or legal reasons.

There are very few other organs such as heart, liver, and eyes which were donated from the South West region.

It is estimated that there is an average of 180 potential donors each year in the South West region, while the actual number of donors is less than 50. Therefore, we should make very effort to triple this number.

### SURGERY IN A COTTAGE HOSPITAL

**J. D. Church, Axminster**

The paper looked at the history of cottage hospital surgery as reflected in Axminster, a small market town some thirty miles from the nearest district general hospitals, where for the past sixty years or more a surgical service of self sufficiency had been carried on. The surgery at present is mostly performed by a surgically qualified general practitioner under the aegis of an Exeter consultant surgeon. Some 300 or so in-patient operations are carried out in Axminster each year such as appendicectomies, operations on varicose veins and piles, sterilisations, vaginal repairs, D & C's and hernias. A report on the hernias done in Axminster over a ten year period was presented with a known recurrence rate over that period of 4%, or about $1\frac{1}{2}$% recurrence rate over five years. An analysis of the recurrence was shown and it was noted that the average age of onset of hernia in our series was aged 54. Perhaps this was an indication of the male menopause! Some 31% of all herniorrhaphies done in the south west region are done in cottage hospitals. The average in-patient stay and average waiting time prior to admission shows wide swings, suggesting that a regional computerised waiting list for cold surgery be introduced.

At present some 70,000 operations a year are carried out in cottage hospitals and it seems that providing there are adequate facilities, enthusiasm, previous experience, attention to detail, acceptance of an audit and consultant involvement there is a good case for expanding this sort of surgery, the by-product being shorter waiting lists, continuity of care, utilisation of married part-time nurses and a very low infection rate. Finally, it is convenient for our raw material – the patient.

### South West Surgical Prize 1984

Seventeen papers were submitted from which the following three papers were selected for oral presentation at the meeting. The prize was awarded to Mr. P. P. Nakak.

### SUCTION DRAINAGE OF WOUNDS AT HIGH AND LOW NEGATIVE PRESSURES

**P. P. Nakak, Torbay Hospital, Torquay**
*Sponsor*: **A. M. N. Gardner**

In a controlled prospective trial in 50 patients undergoing mastectomies, the conventional high negative pressure of 500 mmHg (with respect to atmospheric pressure) applied to the wound drainage was compared with a lower pressure of 160 mmHg of mercury. The lower negative pressure effectively opposed the flaps to the chest wall and it was found that the group subjected to the lower negative pressure had an average of 167±15 ml of fluid drainage from the wound and their stay in hospital lasted on an average 5.3±0.3 days. The corresponding figures for the group receiving the higher negative pressure were 377±422 ml and 8.3±0.6 days respectively. The difference between the mean number of days stay in the hospital for each group was noted statistically using Student's $t$-test and was found to be significant ($t=4.3<0.005$).

It was also noted that the incidence of fluid collection under the flaps after the drains have been removed was higher in high negative pressure suction groups.

It was concluded that the optimum pressure for suction drainage for mastectomy wounds is 160 mmHg.

### THORACOSCOPY IN PLEURAL EFFUSION

**S. A. M. Nashef, Royal Devon and Exeter Hospital, Exeter**
*Sponsor*: **K. M. Pagliero**

Over the past four years 30 patients who presented to the Thoracic Surgical Unit at Exeter with a proven pleural effusion of unknown aetiology were investigated using the technique of thoracoscopy and biopsy under general anaesthetic.

In 27 patients (90%) thoracoscopy yielded a definitive diagnosis which obviated the need for further invasive investigation and allowed treatment to be given where required. In one patient no definitive histological diagnosis could be made at thoracoscopy or subsequent thoracotomy. There were two patients (7%) in whom thoracoscopy failed to demonstrate pleural tumour deposits which became evident subsequently at thoracotomy three and six months later.

Twenty-three patients (77%) had previously been investigated unsuccessfully by bronchoscopy, aspiration cytology, pleural biopsy or a combination of all three procedures; some patients had as many as six attempts at diagnosis using the above procedures before being submitted to thoracoscopy.

There was no mortality associated with the procedure and morbidity was minimal.

We conclude that thoracoscopy is an effective, efficient and safe method of establishing diagnosis in patients with pleural effusion.

### COLOSTOMY CLOSURE – A CLINICAL AND EXPERIMENTAL STUDY

**M. E. Foster, Bristol Royal Infirmary**
*Sponsor*: **D. J. Leaper**

Many risk factors are responsible for the high complication rate following colostomy closure. A study from this region reported 22% anastomotic failure.

From 1975–1979, in Bristol Royal Infirmary, 42 patients underwent colostomy closure with a leak rate of 30%, whereas in the subsequent four years, 71 patients had a leak rate of 10% ($P<0.05$). Closures within one month of colostomy formation leaked in 60% whereas later closure was followed by leaks in 14.5% ($P<0.05$). Closures of Hartmann procedures increased significantly from 32% to 56% ($P<0.01$). Leakage here represented 42% of failures overall.

To examine these findings, 40 rats underwent Hartmann's operation. 20 (A) were reversed at three weeks, 20 (B) at six weeks. Colonic blood flow – (by Laser Doppler) and collagen concentration were measured before anastomosis. Three days later bursting pressure and collagen were measured in the anastomosis. Collagen concentration in Group B was higher in proximal and distal colon before closure; proximal B 15.4 (12.4–20.9) mcg/mg hydroxyproline vs. A, 13.7 (10.4–18.1) ($P<0.05$); distal B 15.9 (7.8–19.9) vs. A, 11.4 (8.7–17.5) ($P<0.02$). Anastomotic bursting pressure was higher in Group B 150 (110–210) vs. A 142 (70–190) mmHg ($P<0.02$). There was a significant correlation between distal pre-closure blood flow and bursting pressure in Group A ($r=0.80$, $P<0.01$).

Anastomotic failure after early closure is related to poor tissue perfusion and low colonic collagen concentration. Clinical results have improved significantly.

